# Non-participants in policy efforts to promote evidence-based practices in a large behavioral health system

**DOI:** 10.1186/s13012-017-0598-4

**Published:** 2017-05-25

**Authors:** Rebecca E. Stewart, Danielle R. Adams, David S. Mandell, Gayatri Nangia, Lauren Shaffer, Arthur C. Evans, Ronnie Rubin, Shawna Weaver, Trevor R. Hadley, Rinad S. Beidas

**Affiliations:** 1 Department of Psychiatry, University of Pennsylvania Perelman School of Medicine, Philadelphia, PA USA; 2 School of Social Service Administration, University of Chicago, Chicago, IL USA; 3 Department of Behavioral Health and Intellectual disAbility Services, Philadelphia, PA USA; 4 Community Behavioral Health, Philadelphia, PA USA; 5 American Psychological Association, Washington, DC USA

**Keywords:** Evidence-based practices, System-level, Policy, Training initiatives, Fidelity

## Abstract

**Background:**

System-wide training initiatives to support and implement evidence-based practices (EBPs) in behavioral health systems have become increasingly widespread. Understanding more about organizations who do not participate in EBP training initiatives is a critical piece of the dissemination and implementation puzzle if we endeavor to increase access in community settings.

**Methods:**

We conducted 30 1-h semi-structured interviews with leaders in non-participating agencies who did not formally participate in system-wide training initiatives to implement EBPs in the City of Philadelphia, with the goal to understand why they did not participate.

**Results:**

We found that despite not participating in training initiatives, most agencies were adopting (and self-financing) some EBP implementation. Leadership from agencies that were implementing EBPs reported relying on previously trained staff to implement EBPs and acknowledged a lack of emphasis on fidelity. Most leaders at agencies not adopting EBPs did not have a clear understanding of what EBP is. Those familiar with EBPs in agencies not adopting EBPs reported philosophical objections to EBPs. When asked about quality assurance and treatment selection, leaders reported being guided by system audits.

**Conclusions:**

While it is highly encouraging that many agencies are adopting EBPs on their own, significant questions about fidelity and implementation success more broadly remain.

## Background

Implementation of evidence-based practices (EBPs) has become an integral focus in behavioral health service delivery [1–3] due to increasing awareness that EBPs are not widely available in community behavioral health settings [4–6]. The implementation of EBPs could confer many advantages to the public sector including better therapeutic outcomes and improved cost-effectiveness compared with treatment as usual [7, 8]. The process of implementing EBPs in public behavioral health systems is complex and fraught with challenges; about half of implementation efforts fail [9, 10].

Policymakers in many large behavioral health systems including Philadelphia, Washington, New York, Hawaii, and Los Angeles County recently have invested significant resources in supporting EBP implementation [11, 12]. Some systems have legislatively mandated that organizations and providers use EBPs (e.g., Washington) or tied payment to the use of EBPs (e.g., Los Angeles). Other systems have supported the use of EBPs by offering EBP training but without mandating their use (e.g., Philadelphia). Understanding factors that lead to participation in voluntary initiatives can provide insight into what drives individuals and organizations to adopt EBPs when not required. Recent studies examine the perspective of stakeholders in large behavioral health systems who participate in EBP training initiatives [13, 14]. Little is known, however, about those that do not participate in such initiatives [15, 16].

For the purposes of this paper, non-participators are defined as those organizations who did not participate in formal system-led training initiatives. Non-participators may include both adopters and non-adopters of EBPs. Gaining a more granular understanding of non-participators can help us identify factors that can be targeted by tailored implementation strategies [17]. It is likely that those that did not participate in EBP training initiatives are a heterogeneous group, and different reasons may drive non-participation. We hypothesize several sub-categories of non-participants, including those (1) who deliberately do not adopt EBPs, (2) who adopt EBPs but without relying on system support, and (3) who are not aware of EBPs or the training opportunities provided. The purpose of the present study was to engage with leaders of organizations that have not formally participated in system-wide training efforts to implement EBP with the goal of understanding the perspectives and experiences of the leaders of these non-participating organizations.

## Methods

### Setting

In Philadelphia, over the past 8 years, the Department of Behavioral Health and Intellectual disAbility Services (DBHIDS) has supported the implementation of several EBPs in select mental health and substance abuse agencies across multiple levels of care. Specifically, DBHIDS has implemented the Beck Community Initiative (cognitive therapy [18, 19]), the Trauma Initiative (prolonged exposure, trauma-focused cognitive behavioral therapy [20]), and the Dialectical Behavioral Therapy Initiative. Agencies receive free training, supervision, and in the case of trauma-focused cognitive behavioral therapy, an enhanced reimbursement rate. In 2012, DBHIDS established the Evidence-Based Practice & Innovations Center (EPIC), a centralized infrastructure that supports implementation and sustainment of EBPs in the community [16]. Approximately 50 of the 200 agencies in the Community Behavioral Health (CBH) network^1^ have participated in one or more EBP training initiatives [16]. The 75% of agencies that are non-participants are the focus of the present study. These EBP training initiatives are part of a larger systemic transformational context that began in 2005, emphasizing and prioritizing the cultures, resilience, and strengths of consumer and their families in a recovery-oriented and trauma-informed framework [20, 21]. The process through which DBHIDS selects organizations for initiatives has evolved over time. Initially, selection was largely guided by DBHIDS (e.g., larger, excelling, and failing organizations were chosen). More recently, organizations have applied for participation through a competitive request-for-applications (RFA) process and are selected for participation by DBHIDS [16]. All procurement opportunities are systematically distributed through the CBH Executive Director listserv. Announcements regarding the EBP initiatives are also disseminated through the (optional) CBH-News and EPIC listservs, and are posted on the DBHIDS, CBH, and EPIC websites.

### Participants and procedure

To obtain a sample of EBP non-participating agencies (NPAs) in this system, we included in our sample all mental health and drug and alcohol treatment agencies located in Philadelphia county who (1) serve more than 100 clients yearly, (2) had not participated in one or more of the DBHIDS initiatives, and (3) had no other known EBP activity (e.g., participation in non-DBHIDS (external but formalized grant funded) EBP trainings, initiatives or implementations), resulting in an eligible sample of 51 agencies. An additional four agencies were eliminated because they closed (2) or were acquired by another agency who was an initiative participant (2).

We contacted agency leadership of each of these 47 agencies via e-mail to ascertain interest. We focused on leaders because we wanted to speak with the person at the agency who would be best acquainted with the selection, operations, implementation, and management of current and new practices. Three agencies declined participation and 11 agencies did not respond to repeated recruitment efforts. Of the 33 agencies who agreed to participate, 30 agencies were interviewed (response rate = 64%; the remaining three agencies were not responsive to scheduling requests after initially agreeing to participate). Interviews were conducted in-person by the first author between May 2015 and January 2016. Participants gave written consent and were compensated $150 for participation.

### Qualitative interview

We developed a semi-structured interview guide consisting of three parts. The first set of questions was exploratory and system-specific: we asked if leadership had awareness or knowledge of the system-sponsored EBP training offerings and if the agency had applied to participate in these initiatives (e.g., What has your agency’s exposure been to the DBHIDS EBP initiatives? Has your agency taken part in the DBHIDS EBP initiatives?). In the second part of the interview, we asked about general attitudes towards EBP and if and how the agency had engaged in or implemented any EBP (e.g., Tell me what you think of when you hear the term EBP? Tell us about any experiences with EBP to date in your agency.). Third, we asked questions to understand more about the agency’s overall treatment philosophy, quality assurance practices, treatment approaches, and population (e.g., What is the agency treatment philosophy or mission? How do you ensure quality of your practices/therapists?).

### Data analysis

Grounded theory informed our overall data analytic strategy given its exploratory nature [22]. An iterative process was used to analyze and develop a codebook for the interview transcripts. The investigators developed a set of codes through a close reading of five transcripts that was applied to the data using an inductive approach [23]. The coders then coded four more transcripts separately and met to adjudicate differences, create additional codes, develop coding rules, consolidate redundant concepts, and finalize the codebook. Coders used NVIVO qualitative data analysis software. Once coding was complete, a random 20% of transcripts were coded by two investigators and the inter-rater reliability was found to be excellent (*κ* = .98; [24]). The first author read through the coded nodes and produced memos including examples and commentary regarding emergent themes within the nodes. Finally, the first and last authors conducted consensus coding. The interview and codebook are available by request from the first author.

A central question to this research was whether the NPAs were adopters or non-adopters of EBP. We coded an agency as an “adopter agency” if the leadership interviewed described his or her agency or at least one therapist as engaging in any practice listed as an EBP on the Substance Abuse and Mental Health Services Administration National Registry of Evidence-based Programs and Practices [25]. Agencies in which leaders did not endorse implementing any EBP reported in this National Registry were coded as “non-adopters.” Based on the transcripts, the first author sorted the 30 agencies into the two categories, and the last author categorized a randomly selected subset (20%) with 100% reliability to the first author.

## Results

### Participants

Characteristics of the leaders and the agencies they represented are denoted in Table 1, column 1. Leaders included 24 executive directors/CEOs and six clinical directors.

**Table 1:** Characteristics of non-participant agencies and leaders

Leader/agency characteristics	Total non-participants (*N* = 30)	Adopters (*N* = 20)	Non-adopters (*N* = 10)
	*n* (SD)	*n* (SD)	*n* (SD)
Leader age	54.82 (12.10)	53.29 (12.80)	57.19 (11.07)
Years at agency	10.34 (8.16)	9.39 (7.33)	11.91 (9.53)
Years at position	7.56 (6.34)	6.87 (6.29)	8.70 (6.56)
Average number of clients served per year by leader’s agency	1049.81 (1030)	929.35 (928.66)	1225.18 (1188.01)
	*n* (%)	*n* (%)	*n* (%)
No knowledge of EBP initiatives	18 (60)	12 (60)	6 (60)
Knowledge of EBP initiatives	12 (40)	8 (40)	4 (40)
Applied/applying to initiatives	4 (13%)	4 (20)	0 (0)

*EBP* evidence-based practice, *NPAs* non-participating agencies

### Knowledge of EBP initiatives

The first question of our investigation is if the NPAs held knowledge about the initiatives. Most NPAs (60%) were unaware of the DBHIDS EBP training initiatives (“never heard of them”) and expressed interest in hearing more information. Of the agencies who knew about the EBP initiative opportunities, 67% had not applied to take part in initiatives due to time constraints associated with participating in the initiative and/or completing the application, or disinterest in the training opportunity. The remainder (13%) had applied to the initiatives and were rejected or in the midst of the application process.

### Adopter/non-adopter categorization

A second question of this investigation is if NPAs were adopting EBPs despite their non-participation in the system-led training initiatives. The results of our EBP adopter categorization are as follows: Of the 30 NPAs, we classified 20 (67%) as EBP adopter agencies and 10 (33%) as EBP non-adopter agencies. Adopters and non-adopters were equally aware of the EBP initiatives, and a significant minority of adopters had applied or were applying to the initiatives. Fisher’s exact tests revealed no differences on the demographics of adopters and non-adopter leaders or agency characteristics (All *p*s > .30; see Table 1).

### Adopter agencies

A list of interventions named by the adopter agencies can be found in Table 2. Adopter agencies represented agencies who were adopting EBP outside of system-led training initiatives. Adopter agencies reported two major themes including a reliance on the prior training and qualifications of their staff in order to practice EBP and a lack of emphasis on fidelity.

**Table 2:** List of treatments reported by adopter agencies

Applied behavioral analysis (ABA)	
Cognitive behavioral therapy (CBT)	
Cognitive therapy (CT)	
Dialectical behavior therapy (DBT)	
Eye movement densensitization and reprocessing (EMDR)	
Family focused therapy (FFT)	
Medication assisted treatment (MAT)	
Mindfulness-based stress reduction (MBSR)	
Motivational interviewing (MI)	
Parenting inside out	
Parent child interaction therapy (PCIT)	
Sanctuary	
Seeking safety	
Social skills training	
Trauma-focused cognitive behavioral therapy (TF-CBT)	
Trauma art narrative therapy (TANT)	
Trauma recovery and empowerment model (TREM)	

#### Competence of staff

Leaders from adopter agencies asserted that they hired only qualified therapists (“professional staff”) with “EBP-oriented attitudes” who acquired training in graduate school. As one supervisor noted, “We only hire masters-level clinicians and I find that that’s (EBPs) what their training has been.” Agency directors acknowledged paying for some training (internal, external, or online) but primarily relying on “teach-backs” to train the rest of the staff. As one described: “We can’t directly have someone like [the University of Pennsylvania] train contractors. That’s impossible, but it’s sort of the Jesus model. Jesus and his apostles train the trainers who all very much commit and go out and proselytize.” Leadership from adopting agencies also noted that securing outside grants to finance trainings was important as was culture change.

#### Fidelity

Most agency leaders reported that fidelity to EBP was not prioritized in their agencies: “We don’t get crazy about fidelity.” Fidelity was often viewed as not feasible in community behavioral health. As one clinical director described: “I try to teach these rigid [cognitive therapy] protocols to my therapists and they say, ‘This won’t fly with my guys.’”

### Non-adopter agencies

Most leaders (60%) at non-adopter agencies required clarification on the definition of EBPs. The remainder were familiar with EBP but had clear philosophical objections.

#### Confusion about EBP

Many equated EBPs with outcomes. These administrators tended to view EBPs simply as a means to an end (i.e., collecting outcomes) rather than a manualized set of clinical practices that have been found to have robust patient outcomes in randomized controlled trials. For example, one leader said, “When I think about EBPs I think of tangible criteria that will measure where a person started, where at midpoint, and where they finally end.”

#### Philosophical objections to EBP

The remaining leaders of non-adopter agencies demonstrated a clear understanding of EBPs but reported philosophical misgivings about EBPs and objections about the practices themselves and concerns about who is promoting these practices and their fit to community populations. One non-adopting CEO epitomized such misgivings when he noted: “I think it’s unethical for CBH, which is after all an insurance company, to be pushing and promoting and putting words in therapists’ mouths about what they should do.”

### Comparisons between non-adopter and adopter agencies

We conducted post hoc thematic comparisons between adopter agencies and non-adopter agencies to identify if thematic differences emerged between the two groups. Differences between the two groups included attitudes about intervention-population fit, treatment approaches, policy-directed care, and quality assurance.

#### Attitudes about fit with population

Leaders from non-adopter agencies were more likely than leaders from adopter agencies to express concerns that EBPs (or manualized treatments) were not applicable to their work in community behavioral health centers, specifically their complex and comorbid client populations: “It [EBP] doesn’t fit the needs of the people we are serving.” Also prominent among non-adopter agency leadership were beliefs that EBPs should be blended with the “art” of therapy and real-life experiences, or that EBPs often do not apply holistically: “Sometimes I have to leave a manual or the theory and work with my gut because the clients I had before me were real clients with real need and need help and I can’t just stick with the therapy.”

In contrast, leaders from adopter agencies were more likely to believe that EBPs fit well with their population. As one remarked: “The [cognitive-behavior therapy] treatment approach seems to be…direct for our population. Some of our population, their average is about 5th or 6th grade reading level. The gentlemen and women that we work with—seem to understand it well.”

#### Treatment modalities

Leaders from non-adopting agencies reported using primarily spirituality and faith-based modalities. As one executive described, “We do the work of walking alongside hurting and broken people and we see that as sacred.” In contrast, leadership from adopter agencies endorsed cognitive behavioral strategies and client-centered and eclectic approaches. As one clinical director explained, “It’s not so unstructured that we look dynamic—as much as I hate to use this term, it’s more eclectic cognitive therapy.”

#### Quality assurance

When queried about treatment selection and overall quality assurance practices at their agencies, a majority of leaders from adopting agencies identified internal mechanisms, such as staff meetings, supervision, and internal and audits as quality assurance practices at their agencies. One director noted, “We firmly believe that in order to provide a service you need to have a highly trained and highly supervised staff who is receiving direct feedback in order to make sure we have treatment integrity.” Non-adopter agency leadership primarily identified external system-level audits as a way to ensure quality: “And really, the audits that CBH has would check on those things [quality].”

## Discussion

The present study is the first examination of community behavioral health agencies that do not participate in system-sponsored EBP training initiatives. This research is relevant and timely given the increasing number of public health settings in which EBPs are encouraged, often with associated resources such as free training and supervision, or an enhanced reimbursement rate. The results from our study indicate that many leaders from NPAs were unaware of the EBP initiative offerings, indicating lost opportunity and that policymakers may need to do more effective outreach to inform all providers of opportunities. Despite not participating in the formal EBP efforts led by the system, many of the NPAs in the Philadelphia system are implementing EBPs on their own accord with their own resources. Despite this promising and somewhat unexpected finding given the fiscal challenges in community behavioral health [13, 26], leaders from adopter agencies unapologetically acknowledged that they did not prioritize rigid fidelity during the implementation process. Maintaining fidelity is resource-intensive, and it is not surprising that other demands in a community behavioral health agency take precedence. Although some research suggests a high level of adherence may not be necessary for successful treatment outcomes (e.g., [27]), the efficacy (as well as acceptability and sustainability) of treatment implementation in the absence of fidelity checks remains an important empirical question.

This lack of focus on rigid fidelity may be best explained by the manner through which adopter agencies gained expertise in EBPs. Leaders from most adopting agencies reported that their agencies were not participating in any formal training, supervision, or consultation efforts, and relied instead on previously trained staff, 1-day workshops, and teach-backs. Although an updated survey is warranted, past surveys of training programs in many disciplines suggest that training programs fail to provide even a minimum level of EBP training [28–30]. It may be problematic to rely on the prior training of an incoming workforce. More research is needed on the efficacy of these training efforts compared with proven effective multi-component training models [31–33]. Nonetheless, adopting agencies must be lauded for implementing complex practices such as EBPs without any systemic support.

Leadership from a number of non-adopting organizations reported not implementing EBP because they view EBPs as incompatible with their agency’s clinical practice. Almost every implementation science framework describes the importance of the fit between the intervention and the population in which it will be implemented [34, 35], and stakeholders consistently raise it as a challenge to the adoption and use of EBP [36–41]. Interestingly, in our prior work with stakeholders in Philadelphia who participated in the EBP training initiatives, we found that leadership did *not* raise intervention/client fit characteristics as a barrier to implementation and were more likely to label the fit between intervention and client as a facilitator [13]. In addition, leadership from adopting organizations in the current study reported that EBPs fit well with their population. Whether adopters and non-adopter agencies serve different clientele remains an empirical question. It is also possible that implementing an EBP may change attitudes about intervention/client fit. Nonetheless, this fundamental difference between adopters and non-adopters in their belief that a particular EBP or EBPs in general is compatible with their clients suggests an important lever for intervention. Continued work is needed with non-adopters to understand these beliefs and perhaps to shift the standard manual or training protocol to fit the realities and needs of front-line service providers and administrators. Although vivid case presentations of research-based treatments hold some persuasive value with clinicians [42], more empirical research is needed on whether focused EBP promotional efforts can change attitudes or translate to training interest at the leadership level. In addition, several non-adopters were unclear about the basic fundamentals defining EBP, indicating different promotional strategies are needed across NPAs.

Although leadership from adopter agencies endorsed the use of internal quality assurance mechanisms, leaders from both adopter and non-adopter agencies saw payer audits and documentation requirements as an acceptable form of quality assurance. This promising finding suggests that agency leadership may find audits helpful towards their goal of maintaining and enhancing quality. Regulators and policymakers therefore have an opportunity to design their audits with the potential to regulate the use of EBP [43–45]. If audits can at least partially achieve the goal of driving clinical practice and quality, perhaps it is time to re-visit the oft-stated axiom that “mandates do not work” to improve EBP implementation. Similar to prior findings [13], our results suggest that clear messaging and prioritizing as part of these mandates is essential for them to achieve their desired effects. More research is also needed on the most effective strategies and channels to educate about and promote EBPs to stakeholders.

Several study limitations should be mentioned. First, our results are based on interview self-report data, and self-presentation bias may be present on behalf of the individual or the organization. Most indicators suggest that administrators were forthright—in fact many seemed eager to share frank opinions. Although our raters achieved perfect reliability, our classification of agencies as adopter or non-adopter agencies was not confirmed with observation or chart review. Recent research cautions that therapist report of the services they deliver may be inaccurate, overestimating the amount of EBP present [46]. An additional limitation of our classification is that agency leaders were not specifically queried on the NREPP registry but probed regarding general practices; we may have missed report of some EBP as a result. This study only included the perspective of agency leadership; it would be interesting to also include the perspective of other stakeholders including therapists and consumers to understand if their perspectives diverge [2, 13]. Our qualitative sample was small, limiting statistical comparisons we can make between characteristics of adopter and non-adopter leaders and agencies. Lastly, our findings are specific to one behavioral health system and factors specific to this system may limit generalizability of the findings.

## Conclusions

The current study lends support to the idea that EBP is diffusing beyond those who are reached through formal initiatives. Despite this promising finding, our results suggest less than optimal training and supervisory conditions with little attention to fidelity, which may lead to compromised outcomes [47]. It is still impressive, however, to note that these agencies have chosen to implement EBP using their own resources. Unlike the cost of undertaking entire initiatives, it may be cost-effective for policymakers to intervene with specific strategies to enhance training, supervision, and fidelity in these “low-hanging fruit” adopting agencies. It is also possible that self-resourcing adopting agencies may endow leadership with more buy-in as compared to those who participate in EBP training initiatives, another empirical question for future research. The present research suggests the need for alternative strategies to educate about and enhance EBP more widely within a system (including consumers), beyond additional training initiatives.

## Abbreviations

CBH: Community Behavioral Health
DBHIDS: Department of Behavioral Health and Intellectual disAbility Services
EBPs: Evidence-based practices
NPA: Non-participating agency

## Footnotes

1 Community Behavioral Health (CBH) is a not-for-profit 501(c)(3) corporation contracted by the City of Philadelphia for provision of behavioral health coverage for the City’s over 500,000 Medicaid-enrolled individuals.
